# Prevalence and management of lower limb segmental overgrowth in patients with NF1: an observational study

**DOI:** 10.1186/s13023-025-04190-5

**Published:** 2026-01-12

**Authors:** Claudia Santoro, Gabriele Martin, Gianluca Conza, Annalisa Itro, Maria Colonnese, Niccolò Garofalo, Giulio Piluso, Gianluigi Federico, Marco Paoletta, Sara Liguori, Antimo Moretti, Silverio Perrotta, Giuseppe Toro

**Affiliations:** 1 Department of Woman, Child, and General and Specialist Surgery, University of Campania “Luigi Vanvitelli”, Naples, Italy; 2 Department of Medical and Surgical Specialties and Dentistry, University of Campania “Luigi Vanvitelli”, Via L. De Crecchio 4, Naples, Italy; 3 Department of Precision Medicine, University of Campania “Luigi Vanvitelli”, Via L. De Crecchio 7, 80138 Naples, Italy; 4 Department of Orthopaedic Surgery, Santobono Pausilipon Children Hospital, Naples, Italy; 5 Department of Mental and Physical Health and Preventive Medicine, University of Campania “Luigi Vanvitelli”, Naples, Italy

**Keywords:** Neurofibromatosis type 1, NF1, Lower limb discrepancy, Overgrowth, Treatment, Quality of life, GOAL-LD, Plexiform neurofibroma

## Abstract

**Background:**

Neurofibromatosis type 1 (NF1) is a neurocutaneous disorder characterized by a potential multisystemic involvement. The musculoskeletal system is frequently affected (i.e.: scoliosis, thorax anomalies, tibial dysplasia). The segmental overgrowth of lower limb (SOLL) is rarely reported, albeit it severely affects patients’ wellbeing. The resulting leg length discrepancy (LLD) negatively impacts the development of the musculoskeletal system and may require appropriate correction, which could include surgery. Our objective was to evaluate the prevalence, the management, and the outcomes in patients with SOLL and NF1. We retrospectively evaluated 553 pediatric patients seen between 1992 and 2024 with a diagnosis of NF1. All patients presenting with SOLL were included in the study. For each patient, we assessed the degree of LLD at the initial evaluation, at the time of surgery (if any), at the point of maximum discrepancy, and at last visit. Demographic data, associated deformities, presence and location of plexiform neurofibroma (PN) were registered. Each patient and/or their parents were also evaluated using the Gait Outcome Assessment List for lower-limb differences (GOAL-LD) questionnaire to assess the health-related quality of life of pediatric patients with LLD.

**Results:**

7 patients (4 males) with a mean age at diagnosis of SOLL of 4.67 years met our inclusion criteria. The detected discrepancies ranged from 0.5 cm to 6 cm (mean 4.30). Five children underwent surgery for the discrepancy (3 with epiphysiodesis and 2 with external fixation limb lengthening). All patients but one presented a plexiform neurofibroma in the district of SOLL that has been treated by selumetinib in 3 cases. Two patients were treated conservatively using lifted insoles. The GOAL-LD questionnaire revealed low scores in all the domains evaluated (function and mobility, pain and fatigue, physical and recreational activity, gait appearance, use of braces and walking aids, body image, and self-esteem).

**Conclusions:**

SOLL-related LLD in patients with NF1 is a complex condition with significant impact on quality of life. Outcomes are often suboptimal, underlying the need for individualized, multidisciplinary management and structured follow-up. Early detection of progression is crucial to guide timely therapeutic decisions. Further prospective, multicenter studies are needed to better clarify pathogenic mechanisms and to develop standardized treatment protocols.

## Background

Neurofibromatosis type 1 (NF1) is an inherited disease considered to be one of the most common autosomal dominant disorders, with an estimated prevalence between 1 and 3,000 to 1 in 4,000 patients [1]. It is commonly caused by a heterozygous mutation in the NF1 gene located on 17q11.2 chromosome region, that encodes for the neurofibromin protein, a multifunctional regulatory protein of several processes (i.e., regulation of cell proliferation and differentiation through the RAS/MAPK and RAS/PI3K/AKT signal transduction pathways; regulation of the cell cycle progression interfering with the cAMP/protein kinase A pathways; regulation of the intracellular transport) [2].

NF1 presents a variable phenotype anyway, the main clinical manifestations of NF1, which represent some of the fundamental diagnostic criteria include: café au lait macules, neurofibromas, skin-fold freckling, Lisch nodules of the iris and bone dysplasia [3] classically involving the long bones and sphenoid wing [4].

Segmental lower limb overgrowth syndrome (SOLL) is a rare and likely underestimated manifestation of NF1 characterized by soft tissue hyperproliferation associated with abnormal bone growth, compared to the contralateral limb, leading to potentially severe lower limb discrepancy (LLD).

In the available literature, segmental limb overgrowth associated with NF1 were generally reported along with other overgrowth syndromes. However, these syndromes should be more appropriately considered as unique entities, considering their different natural history, prognosis and management [5]. Therefore, we conducted a retrospective observational study with the following objectives: (1) evaluate the prevalence of SOLL in NF1 children followed at a referral centre; (2) report the natural history and the impact on quality of life of SOLL in NF1; (3) evaluate the efficacy of the commonest way of treatment of SOLL dependent LLD in NF1.

## Methods

We retrospectively reviewed clinical notes of 553 children affected with NF1 and followed at the NF1 pediatric referral centre of the University of Campania “Luigi Vanvitelli” between 1992 and 2024. As previously reported [6], our NF1 care unit is composed by a multidisciplinary team coordinated by two pediatricians which involves several specialists with expertise on NF1 who are engaged on the basis of patient’s specific needs and indicated screening. Questionnaires exploring quality of life, motor abilities and pain are routinely offered to children and their parents or caregivers to better document the general status of the patient and evaluate the impact of a specific complication on daily life and psychological wellness. The diagnosis of NF1 was established according to both National Istitute of Health (NIH) and Legius et al. criteria [3, 7], moreover a genetic test for delineating the NF1 genotype was also performed since 2000.

**Fig. 1:**
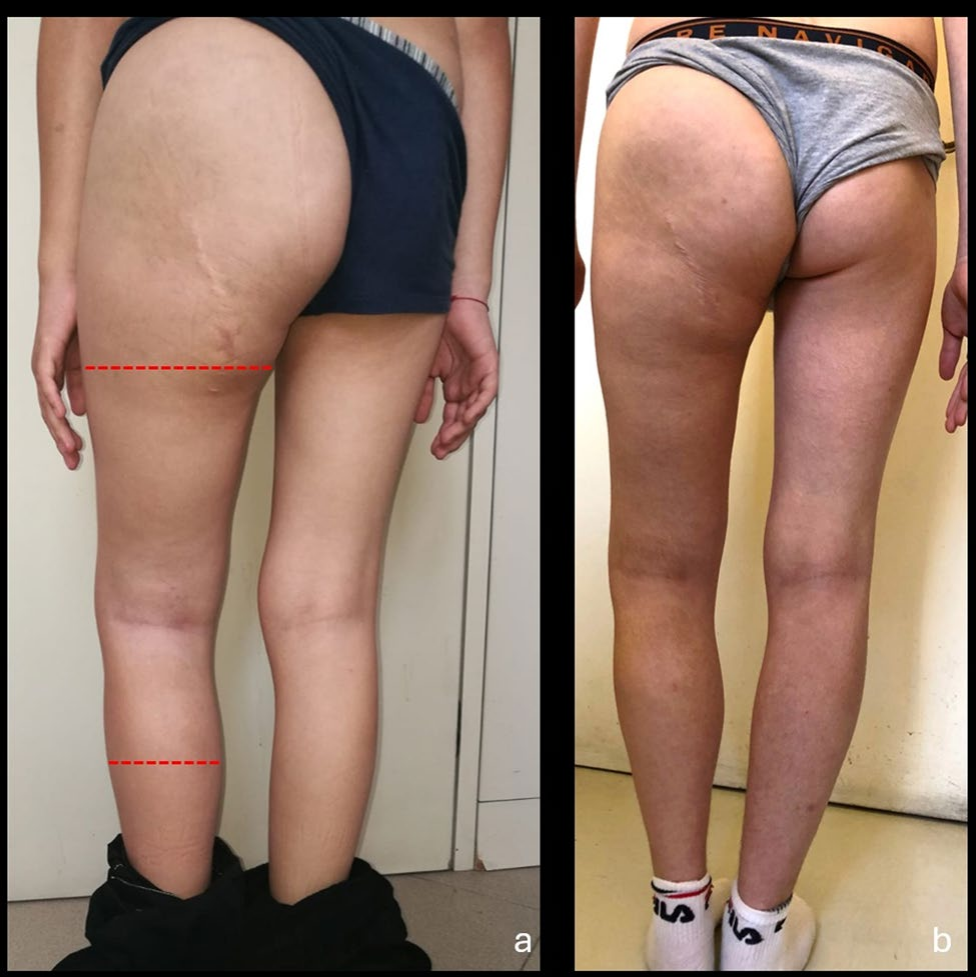
Clinical appearance of the SOLL ad the lumbosacral PN of the patient #2 before (**A**) and after (**B**) pharmacological and surgical treatment of the overgrowth. In red the reference lines for the clinical evaluation of SOLL

The clinical notes of each patient were reviewed using a specific designed evaluation grid. As a protocol, patients were regularly assessed for LLD by the disease manager. If LLD was documented the patient had an orthopedic consultation and x-rays if indicated. In these cases the occurrence of a SOLL was furtherly investigated to confirm or exclude. Considering that a clear definition of SOLL is not available, we defined it as an excessive proliferation of organs or tissues of the lower limb associated with or without bone alterations [8]. The diagnosis of SOLL was conducted by agreement between the case-manager and the orthopedic, comparing both the mid-thigh and mid-leg circumference with the unaffected side (see Fig. 1).

Patients with incomplete clinical history or lost to follow-up were excluded.

The diagnosis of LLD was made through a careful clinical examination with the patient both in supine position and in orthostatism and then confirmed radiographically. If needed, an ultrasonography or magnetic resonance imaging evaluation of the affected limb was also performed to detect/characterize associated plexiform neurofibromas (PN).

Only those X-rays correctly conducted according to the parameters listed in Table 1 were considered. For each patient, we collected demographic data, associated skeletal deformities (i.e.: scoliosis and lower limb malalignment), presence and site of PNs. Moreover, the LLD at first follow-up, the maximum LLD, type of treatment, LLD at the time of surgery (if any), and the LLD at last follow-up were also collected. Finally, when available the type of inheritance and genetics were reported.

**Table 1:** Parameters evaluated to assess a full-length lower limb x-ray as properly conducted

✓ With well-extended limbs (knee not flexed or forced in varus or valgus)
✓ Patella in the frontal plane
✓ Visualising the heads of the femurs and ankles on the same X-ray ✓ Visualising the iliac crest and triradiate cartilages
In cases of LLD greater than 3 cm, an elevation under the shorter limb was used.

**Abbreviations**: LLD, Lower Limb Discrepancy

To evaluate the effectiveness of the proposed treatment and quality of life of the included patients, we administered to them and/or to their parents, The Gait Outcome Assessment List for lower-limb differences (GOAL-LD) [9]. This questionnaire is specifically conceived to evaluate the health-related quality of life in pediatric patients with LLD also in case of neurological disorders.

The GOAL-LD questionnaire covers 7 domains (A to F): Gait Function & Mobility; Pain/Discomfort/Fatigue; Physical Activities, Games & Recreation; Gait Appearance, Use of Braces and Assistive Devices; Body Image & Self-Esteem.

A total of 50 items is submitted to the patients or care givers.

According to the Dermott et al., domains related to tasks or activities use a 7-point ordinal scale ranging from 0: “extremely difficult/impossible” to 6: “no problem at all,” with a 4-point modifier indicating the level of assistance required, from 0: “total” to 3: “independent,” to complete each task or activity [9]. Symptoms such as pain or fatigue are evaluated using a 6-point frequency scale, from 0: “every day” to 5: “none of the time,” along with their intensity, rated on a scale from 0: “severe” to 2: “mild.“ [9]. Domains assessing the respondent’s feelings use a 5-point ordinal scale, from 0: “very unhappy” to 4: “very happy.“ [9]. Domain scores are calculated as the average of the standardized item scores for each item within the domain. The total score is the average of all standardized item scores, reported on a scale from 0 to 100 [9]. The scores for each domain were calculated at final follow-up.

The patients included were divided into two groups based on the treatment received (surgical vs. conservative). The present study complies with the Declaration of Helsinki, revised in 2008. All patients signed a written informed consent, authorizing data collection for research and audit purposes. Data included in the present study were collected according to our institutional ethical approval (Prot. 0009133/i).

Descriptive statistics were used to describe the patients included.

## Results

**Fig. 2:**
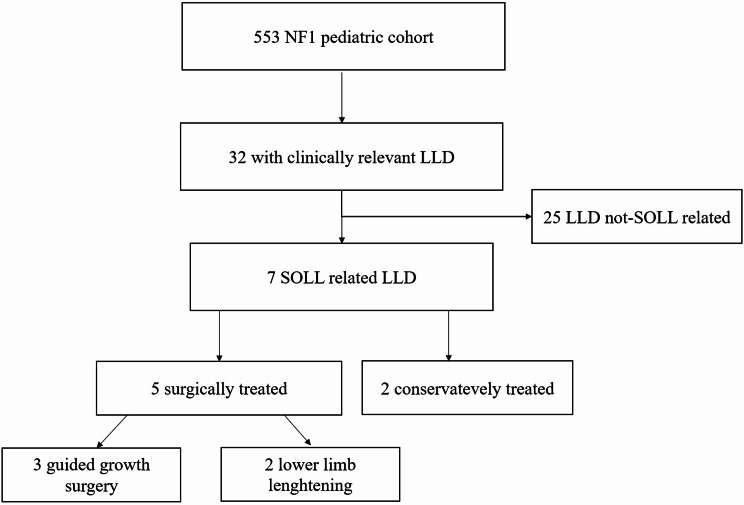
Patient’s selection process

Among 553 clinical reports of patients with NF1, we identified 7 cases (4 males, 3 females) affected by SOLL, accounting thus for a prevalence of 1.3% of the entire studied population. Figure 2 summarizes the patient’s selection process.

The mean age at NF1 diagnosis was 4.67 years. The two groups (Surgical Vs Conservative) did not differ in terms of gender distribution. However, the mean age at diagnosis of SOLL-related LLD was lower in the surgically treated group compared to the conservatively one (5.20 vs. 11.00 years).

All patients but one presented a plexiform neurofibroma on the ipsilateral side of the limb affected by the SOLL. In two cases the PN originated by lumbosacral nerves, deeply, extended to the pelvis, exit through the obturator foramen and involved all the nerves of the lower limb. In one case the patient had a diffuse superficial PN of the leg and ankle with associated oedema. One girl had a superficial PN of the foot and lower leg. The PN was treated with selumetinib in 3 cases with one partial response (patient n.2), one progression after a 1 year of stable disease (patient n.4), one case with stable disease (patient n.1). The first two patients have been recently reported in a small case series given the dramatic effect of the drug on pain due to the PN [10, 11]. In all patients the selumetinib was started when the SOLL had already developed.

Valgus alignment of the lower limbs was more often observed in the surgically treated group (5 surgically treated VS 0 conservatively treated). Scoliosis was observed in both groups (3 surgically treated VS 2 conservatively treated).

See Tables 2 and 3 for further details.

**Table 2:** Characteristics of the included population

Id	Sex	Age at NF1 diagnosis	Lower limb malalignment (y/*n*)	Lower limb malalignment type	Neurofibroma on the affected limb (y/*n*)	LLD at t0 (cm)	Age at t0 (yrs)	Max LLD (cm)	Age at max LLD (yrs)	LLD at surgery (cm)	Age at surgery (yrs)	LLD at last follow up (cm)	Age at last follow up (yrs)	Associated scoliosis (y/*n*)	Surgical treatment (y/*n*)	Type of treatment
1	F	8,00	Y	Genu valgus	Y	1,00	8,00	5,00	16,00	5,00	16,00	0,00	17,00	N	Y	Lower limb lengthening
2	M	11,00	Y	Genu valgus	Y	6,00	11,00	6,00	11,00	6,00	12,00	-0,50	17,00	Y	Y	Guided growth
3	M	4,00	Y	Genu valgus	Y	2,00	4,00	5,50	12,00	5,50	8,00	5,00	13,00	Y	Y	Guided growth
4	F	3,00	Y	Genu valgus	Y	3,00	3,00	5.5	9,00	5.5	11,00	-1,00	18,00	Y	Y	Lower limb lengthening
5	F	2,00	N	N/A	Y	0.5	13,00	0.5	24,00	N.A.	N.A.	0.5	24,00	Y	N	Raised heel insole
6	M	7,00	Y	Genu valgus	Y	3,00	8,00	3,00	11,00	3,00	11,00	2,00	11,00	N	Y	Guided growth
7	M	1,00	N	N/A	Y	2,00	12,00	2,00	12,00	N.A.	N.A.	2,00	20,00	Y	N	Raised heel insole

**Abbreviations**: F, Female; M, Male; LLD, Lower Limb Discrepancy; N/A, Not Available

**Table 3:** Difference between groups of the analyzed population

	NF1 Cohort (*n* = 553)	Whole population with SOLL (*n* = 7)	Surgically Treated Group (*n* = 5)	Conservative Treated Group (*n* = 2)
Sex (F)	239	3	2	1
Mean age at NF1 diagnosis (yrs)	N/A	4,67; +/- 3,72	6,25; +/- 3,59	1,50; +/- 0,71
*De novo* NF1	310	3	2	1
Familial NF1	282	4	3	1
Maternal NF1	145	2	2	0
Paternal NF1	114	2	1	1
Unspecified	23			
Associates scoliosis (*n*)	54	5	3	2
Genu Valgus Malalignment (*n*)		5	5	0
Left (*n*)		2	2	0
Right (*n*)		2	2	0
Both Sides (*n*)		1	1	0
Plexiform neurofibroma on affected limb (*n*)		7	5	2
Overgrowth Side				
Left (*n*)		3	2	1
Right(*n)*		4	3	1
LLD at T0 *cm* (mean; ds)		2,83 ; +/- 1,72	2,50 ; +/- 1,87	2,50 ; +/- 0,71
Age at T0 *yrs* (mean; ds)		8,43 ; +/- 3,87	5,67 ; +/- 3,27	11,00 ; +/- 2,65
Max LLD *cm* (mean; ds)		4,30; +/- 1,72	3,90 ; +/- 1,31	2,50 ; +/- 0,71
Age at max LLD *yrs* (mean; ds)		13,57; +/- 5,06	9,83 ; +/- 2,59	15,67 ; +/- 7,23
LLD at surgery time *cm* (mean; ds)		4,88 ; +/- 1,31	3,90; +/- 1,31	
Age at surgery time *yrs* (mean; ds)		11,60; +/- 2,88	9,67 ; +/- 2,88	
LLD at last follow up *cm* (mean; ds)		0,92 ; +/- 2,23	0,92 ; +/- 2,46	2,00 ; +/- 0,00
Age at last follow up *yrs* (mean; ds)		17,14 ; +/- 4,30	12,67 ; +/- 3,03	22,00 ; +/- 6,66

**Abbreviations**: F, Female; YRS, Years; SOLL, Segmental Lower Limb Overgrowth Syndrome; LLD, Lower Limb Discrepancy

The GOAL-LD questionnaire, whose results are summarized in Table 4, was completed by 5 out of 7 patients, all belonging to the surgically treated group. In these patients the questionnaire was administered at a mean of 27.6 months after the surgery (range 6–60).

**Table 4:** GOAL-LD questionnaire results

Id	Sex	LLD At Last Follow Up (cm)	Age At Last Follow Up (years)	Gait Function And Mobility (A)	Pain, Discomfort And Fatigue (B)	Physical Activities, Games & Recreation (C)	Gait Appareance (D)	Use Of Braces And Assistive Devices (E)	Body Image & Self Esteem (F)
1	F	0	17	752/1100 (68%)	500/600 (83%)	17/100 (17%)	33/500 (6,60%)	0/100 (0%)	0/800 (0%)
2	M	-0,5	17	499/1100 (45%)	240/600 (40%)	167/500 (33%)	67/500 (13%)	25/400 (6,25%)	125/800 (16%)
3	M	5	13	836/1100 (76%)	300/600 (50%)	67/100 (67%)	84/500 (16%)	100/100 (100%)	225/800 (28%)
4	F	-1	18	1018/1100 (92%)	520/600 (87%)	33/100 (33%)	367/500 (73%)	100/100 (100%)	625/800 (78%)
6	M	2	11	767/1100 (74%)	600/600 (100%)	317/400 (79%)	200/500 (40%)	0/200 (0%)	625/800 (78%)

**Abbreviations**: LLD, Lower Limb Discrepancy; F, Female; M, Male

However, 3 patients scored below 33%; while 2 scored 67 and 79% in domain (C), (Physical Activities, Games & Recreation). 4 out of 5 patients reported scores below 40% on items within Domain (D) (Gait Appearance), while the last 73%.

Regarding Domain (E), (questionnaire that evaluates the utilization of orthosis and aids and the patients’ satisfaction with their use), three patients needed a shoe lift, one patient used a foot-ankle orthosis and crutches, and another combined a shoe lift with a foot-ankle orthosis and crutches.

2 out of 5 patients reported a score of 0, while one scored 6.25%, reflecting anyway considerable discomfort associated with these aids. The other two patients achieved a score of 100%, indicating that the use of walking aids and orthoses did not negatively affect their quality of life.

**Fig. 3:**
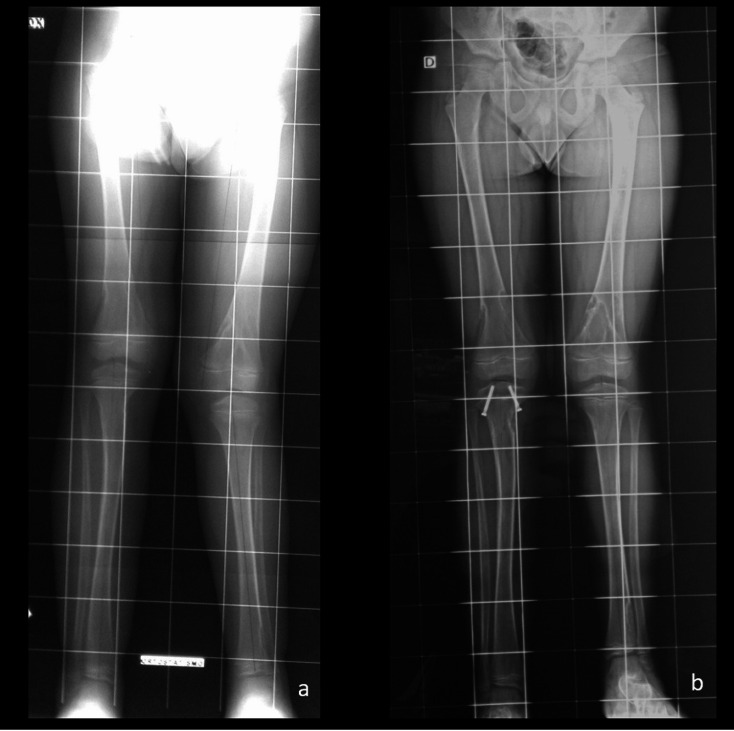
An example of guided growth (patient id# 3). In **A**), full-length lower limb x-rays at time of the surgery showing a genu-valgus and a 5.5 cm of LLD. In **b**), full-length lower limb x-rays at 9 months after the surgery, showing a redution at 3 cm of LLD. Unfortunately, a LLD rebound was observed at screws removal (not showed)

Lastly, regarding Domain (F) (Body Image and Self-Esteem), 3 out of 5 patients expressed dissatisfaction with their physical appearance and personal image because of the asymmetry of their lower limbs (scoring below 30%); while the other two did not feel any significant dissatisfaction with their appearance due (scoring 78%). Figure 3 gave an example of a patient treated through guided growth technique.

Finally in Table 5 we summarized the genetics of the studied population.

**Table 5:** Inheritance of NF1 and pathogenetic variants reported according to NM_000267.3 referral sequence of NF1 gene and their effect of the included children

ID	Inheritance pattern of NF1	DNA and proteic effect of the PV
1	Pat	c.1721G > C p.A548LfsX12
2	Dn	c. 1198 C > T, p.Gln400Ter
3	Dn	c.1423del, p.(Lys476Asnfs*22)
4	Mat	c.1246 C > T. p.Arg416Ter
5	Dn	chr17:29528489 [NM_001042492.3) c. 1246 C > T, p.(Arg416Ter)
6	Mat	c.5839 C > T, p.(Arg1947Ter)
7	Pat	: c.128T > C P.Leu43Pro)

Abbreviations: F, Female; M, Male; **Dn**, de novo; Mat, Maternal; Pat, paternal

## Discussion

### Epidemiology of SOLL in NF1

Little is known about the prevalence of segmental overgrowth of the lower limbs in both syndromic and non-syndromic patients. In fact, they could easily be misdiagnosed because minor asymmetry often are considered normal [12]. However, some data were reported for rare disease [13]. Some data on hemihypertrophy have been published in the past ranging between 10 in 860,000 to 1 in 3000 depending on the underlying disease and geographical area considered [14–16] The true prevalence of SOLL in general and of SOLL in patients with NF1 is still unknown. This is primarily due to the limited number of studies on this issue and the inclusion of patients with NF1 along with other LLD syndromes. To the best of our knowledge our study was the first specifically designed to evaluate SOLL-related LLD in NF1 patients, and in our population the prevalence of this condition was 1.27%. Although this seems quite rare, this prevalence is not so far from other bone dysplasia or dystrophies associated with NF1. For example, the prevalence of a scoliosis in NF1 is about 9.8%, yet it drops down to 3.9% considering only the dystrophic scoliosis (namely those associated with vertebral dysplasia) [17]. Moreover, the prevalence of tibial dysplasia was reported to be approximately 5% [17], while sphenoidal alteration 4% [18].

### Overgrowth pathogenetic mechanism

The mechanism by which patients with NF1 develop a SOLL is not fully understood. Like other dystrophic features observed in NF1, SOLL - related LLD can occur in patients regardless of gender or hereditary background. Two types of overgrowth could be identified in NF1: global and segmental.

Regarding the former, it has been reported in patients with 17q11.2 microdeletion [18]. Douglas suggested that the haploinsufficiency of *RNF135*, which encodes a widely expressed 432–amino acid protein, may contribute to this phenotype. *RNF135* is an *NF1*-adjacent gene included in the typical 17q11.2 microdeletion. Its loss may contribute to tall stature, macrocephaly, and specific facial phenotypes. Notably, the generalized overgrowth described in these cases was of mild to moderate severity and had a postnatal onset [18].

The second form of overgrowth seen in NF1, the segmental one, bears resemblance to that observed in the phosphatidylinositol 3-kinase (PIK3CA)-Related Overgrowth Spectrum (PROS) that is caused by activating somatic mutations in the *PIK3CA* gene, leading to a mosaic pattern of overgrowth. This model is characterized by significant variability in both tissue involvement and anatomical location. Reported manifestations include isolated macrodactyly, hemimegaloencephaly, and localized overgrowth of the feet, hands, or limbs and the affected tissues may include adipose, muscular, neural, and bone [19].

Although the phenotype of overgrowth in PROS patients appears like that observed in NF1, the two conditions should not be considered a single, neither overlapping, clinical entities. One hypothesis was suggested by Sabbioni G. et al. The authors reported a case report of a 3-year-old child with NF1 affected by “lower limb gigantism,” hypothesizing that the presence of a PN was associated with the abnormal growth of the body segments related to the disease [20].

The association between PN and segmental overgrowth was also hypothesized by Bano S. et al. and Ponti et al. in two other case reports, referring to this condition as “Elephantiasis Neuromatosa” [21, 22]. Another possible mechanism is secondary lymphedema, which could drive the transformation of lymphatic fluid into adipose tissue, thereby contributing to volume increase [22].

The strongest hypothesis is that a somatic event, contributing to the pathogenesis of the PN could also lead to segmental overgrowth. In analogy with PROS, the timing of the somatic mutation—or “second hit”—may influence the extent of the phenotype, potentially leading to either isolated overgrowth or more complex presentations such as SOLL associated with PN. This link between PN and SOLL-related limb length discrepancy in NF1 raises important clinical questions, particularly regarding the potential for pharmacological interventions (e.g., MEK inhibitors such as selumetinib) to prevent or limit overgrowth progression. Considering that in all our patients under selumetinib the treatment started after the SOLL had already developed, we were not able to specifically address this effect. Accordingly, we believe that upcoming studies on MEK inhibitors should also address the natural history of SOLL. Timely initiation of therapy may prevent or mitigate SOLL and could ultimately reduce the necessity for surgical procedures.

Lastly, the NF1 genetic background in our patient cohort reflects the typical mutation spectrum observed in the broader NF1 population.

### Management of SOLL in NF1

A LLD can potentially have a negative impact on musculoskeletal biomechanics and may require appropriate correction. Moreover, during childhood, the appropriate surgical indication and timing may be quite challenging [23].

In our series, 2 patients were conservatively treated using heel lifts presenting a LLD of ≤ 2 cm.

Although, there are no data in the available literature directly comparing the effectiveness of conservative versus surgical treatment for LLDs < 2 cm, generally shoe lifts are suggested in these cases to prevent spinal deformities, back pain, and postural changes [24–26]. However, a clear cut-off for conservative treatment is not available. In fact, Gurney et al. suggested the use of internal shoe lifts for LLD up to 20 mm [12], Reid D.C. & Smith B., up to 10 mm [24], while Moseley C.F. up to 60 mm [25]. Unfortunately, we can’t draw a conclusive indication on the clinical effectiveness of conservative treatment in SOLL because both our patients died for reasons not related to the present study before completing the GOAL-LD questionnaire (brainstem tumors).

Five out of 7 patients of our cohort (71%) were surgically treated. The decision to perform surgical treatment was based on both the severity and evolutive nature of SOLL-related LLD. Our surgically treated patients had a mean of 3.9 cm of LLD at the time of the surgery. Based on the biomechanical effects of LLD on musculoskeletal disorders (lower back pain, gait and posture abnormalities, stress fractures, functional scoliosis, hip and knee pain, and joint contractures) a value between 2 and 2.5 cm of LLD is generally accepted as a reasonable threshold for performing surgery [26].

Regarding the surgical procedure, 3 out 5 were treated with growth modulation techniques (epiphysiodesis), while the remaining 2 underwent to limb lengthening using the Ilizarov external fixator.

Guided growth technique is based on the application of fixation devices over an open physis. This technique (also known as temporary epiphysiodesis) exploits the proprieties of decreased growth activity when a physis is subjected to compressive forces [27]. Several studies recommend epiphysiodesis for LLD between 2 and 5 cm [27, 28]. However, in 2/3 of our patients the LLD was greater than 5 cm at the time of the surgery. The choice of performing a guided growth technique also in these patients was related to the presence of open physis and the correction potential associated with modern techniques [29]. The results of both patients were satisfactory in terms of lower limb correction.

To the best of our knowledge our study was the first using the GOAL-LD questionnaire to evaluate the quality of life in SOLL-related LLD. In fact, all patients in the surgically treated group completed the GOAL-LD questionnaire. Mostly of the results reported by our cohort were unsatisfactory, regardless the correction obtained. Particularly, quality of life was mostly affected by their lower limb image and gait pattern which often required the use of walking aids, both of which influenced their external appearance. In fact, 2/5 patients scored zero in the domain E (Use of Braces and Walking Aids), reflecting a severe discomfort and unhappiness, despite the satisfactory surgical correction.

In our opinion the routine use of the GOAL-LD questionnaire might be useful to improve the evidence around LLD managements especially in rare disease like SOLL-related LLD.

### Limitations of the study

To the best of our knowledge, this is the first study specifically conceived on SOLL in patients with NF1. However, the retrospective nature and the absence of direct evaluation of radiographic images might underestimate the true degree of LLDs and their evolution, as well as the prevalence of SOLL. Nevertheless, the regular and scheduled follow-up expected from our multidisciplinary approach makes us confident on the quality of clinical notes. Moreover, it gave us the possibility to track the progression of LLDs. Another limitation was the small number of patients included that might underpowered the statistical analysis. However, the reduction of the inclusion criteria was necessary to limit sampling errors.

## Conclusions

SOLL-related LLD in patients with NF1 represents a complex condition that requires a careful and personalized clinical approach. Our experience showed that although appropriately indicated, the surgical outcomes are often unsatisfactory. This highlights the need for an early diagnosis to perform an early and mini-invasive treatment. It is essential that follow-up is structured and regular to detect any early worsening and promptly guide diagnostic and therapeutic decisions. Although the rare incidence SOLL-related LLD in NF1, the quality of life can be severely undermined, as supported by the results of the GOAL-LD questionnaire especially in terms of social participation and physical function. Further prospective and multicenter studies are necessary to better understand the underlying pathogenic mechanisms and establish effective integrated protocols to improve clinical outcomes in the management of SOLL-related LLDs in patients with NF1.

## Data Availability

The datasets used and/or analyzed during the current study are available from the corresponding author on reasonable request.

## Abbreviations

NF1: Neurofibromatosis type 1
SOLL: Segmental lower limb overgrowth syndrome
LLD: Lower limb discrepancy
PN: Plexiform neurofibroma
GOAL-LD: Gait Outcome Assessment List for lower-limb differences
PROS: Phosphatidylinositol 3-kinase Related Overgrowth Spectrum
F: Female
M: Male
